# Immune Biomarkers in Blood from Sarcoma Patients: A Pilot Study

**DOI:** 10.3390/curroncol29080441

**Published:** 2022-08-05

**Authors:** Sarmini Munisamy, Ammu Kutty Radhakrishnan, Premdass Ramdas, Priscilla Josephine Samuel, Vivek Ajit Singh

**Affiliations:** 1 National Orthopaedic Centre of Excellence in Research and Learning (NOCERAL), Department of Orthopaedic Surgery, Faculty of Medicine, University of Malaya, Kuala Lumpur 50603, Malaysia; 2 Jeffrey Cheah School of Medicine and Health Sciences, Monash University Malaysia, Sunway 47500, Malaysia; 3 Division of Applied Biomedical Sciences and Biotechnology, School of Health Sciences, International Medical University, Kuala Lumpur 57000, Malaysia

**Keywords:** CD4^+^ T-cells, TNF-α, IFN-γ, biomarkers

## Abstract

The main role of the host immune system is to identify and eliminate cancer cells, which is a complex process, but it is not a fail-safe mechanism. Many sarcoma patients succumb to this disease despite treatments rendered. The aim of this pilot study was to compare the levels of CD4^+^ T-cells, T-regulatory (Treg) cells, and cytokines such as tumor necrosis factor-alpha (TNF-α), interferon-gamma (IFN-γ), interleukin-17A (IL-17A), and transforming growth factor-beta-1 (TGF-β1) in peripheral blood leukocytes of sarcoma patients and healthy controls. For gene expression studies, total ribonucleic acid (RNA) was extracted from peripheral blood leukocytes and genes that were differentially regulated in peripheral blood leukocytes of sarcoma patients compared with healthy controls were determined using a commercial T-helper cell differentiation quantitative polymerase chain reaction (qPCR) array. Flow cytometer analysis was performed on blood samples from 26 sarcoma patients and 10 healthy controls to identify the levels of CD4^+^ T-cells and T-reg cells. The level of cytokines in plasma and culture supernatant were quantified using commercial enzyme-linked immunosorbent assay (ELISA) kits. A marked reduction in the percentage of CD4^+^ T-cells (*p* = 0.037) and levels of TNF-α (*p* = 0.004) and IFN-γ (0.010) was observed in sarcoma patients. Gene expression analysis showed five genes (homeobox A10 *(HOXA10),* GATA binding protein 3 *(GATA3),* prostaglandin D2 receptor 2 *(PTGDR2),* thymocyte selection associated high mobility group box *(TOX)*, and C-C motif chemokine receptor 3 *(CCR3)*) were dysregulated (*p* < 0.05) in sarcoma patients. This study suggests that T-helper-1 immune responses are reduced in sarcoma patients.

## 1. Introduction

The immune system is a complex network involving cells and soluble substances, which protects the host from many assaults, including pathogenic microorganisms and tumors [1]. An effective defense mechanism is achieved by regulating both innate and adaptive arms of the immune systems via complex signaling networks [2]. The host immune system plays a role in preventing the establishment of cancers by detecting, recognizing, and destroying cancer cells through a process known as immune surveillance [3]. However, various factors regulate anticancer immune responses by the host immune system. Some factors facilitate anti-cancer effects, allowing recognition and killing of cancer cells, whereas other factors inhibit activation of the host immune system, which hinders the body system’s proper functioning. This dual facet of the host immune system helps to balance the activation of the anti-cancer immune system and tumor establishment. In addition, the interplay between cancer cells, normal stromal cells, and host defense mechanisms can also give rise to tumor development and survival [4]. Cancer cells evade the immune system using an immune escape mechanism known as immunoediting [5]. When tumor suppressor mechanisms fail, tumor cells with reduced immunogenicity can escape the host immune system. In addition, the host immune system will need to overcome the immunosuppressive microenvironment that is often observed in many tumors [4].

The adaptive immune system plays a vital role in preventing cancer development and progression, primarily through T-lymphocytes, B-lymphocytes, and natural killer (NK) cells [6]. Upon activation, the adaptive immune system increases receptor variation that can better identify antigens [7]. Cytotoxic T-lymphocytes (CTL) or CD8^+^ T cells play a crucial role in in recognizing and killing tumor or abnormal cells [8]. Another T-cell subset, CD4^+^ T cells play a pivotal role in the adaptive arm of the immune system to regulate infections, transplantations, autoimmune diseases, and cancer development [9]. CD4^+^ T-cells are broadly divided into T-helper (Th) or T-regulatory (Treg) cells [9]. Th cells can be further divided into T-helper 1 (Th1) [10], T-helper 2 (Th2) [11], T-helper 9 (Th9) [12], T-helper 17 (Th17) [13], T-helper 22 (Th22) [14], and T-follicular helper (Tfh) [15]. Th1 cells promote cell-mediated immunity [16] via production of interferon-gamma (IFN-γ), tumor necrosis factor-alpha (TNF-α), interleukin-2 (IL-2), and interleukin-12 (IL-12) [17], which play a substantial role in recognizing cancer cells and inhibiting their progression by activation of CTL, macrophages [18], and NK cell activity, and upregulation of major histocompatibility complex (MHC) expression on antigen-presenting cells (APCs) [17]. Th1 and CTL cells also regulate host immune response by producing IFN-γ [4]. In contrast, Treg cells function as immune suppressors by inhibiting the activation of both CD4^+^ and CD8^+^ T cells [19], are generally found in higher numbers in tumor microenvironments, and secrete cytokines such as transforming growth factor-beta-1 (TGF-β1) [4]. There is evidence to support the protective role played by TGF-β1 in the early stages of cancers. However, in more advanced cancers, TGF-β1 exhibits tumor-promoting activities [20]. For instance, TGF-β1 can inhibit production of IFN-γ by Th1 and CD8^+^ T-cells, thereby promoting proliferation of Treg and Th17 cells, which support tumor growth and progression [19]. Th17 cells in tumor microenvironment are known to inhibit proliferation of CD4^+^ Th1 [19]. Interleukin-17A (IL-17A) is a pro-inflammatory cytokine produced mainly by Th17 cells [21] and to a lesser extent by CD8^+^ T-cells that express the gamma-delta (γδ) T-cell receptors (TCR) and natural killer T (NKT) cells [22]. This cytokine favors tumor development in cancers by promoting vascularization [23], and its anti-tumor activity is associated with initiation of CTLs, natural killer (NK) cells and neutrophils, stimulation of IL12 secretion by macrophages, dendrite cell maturation, and T-cell priming [17].

This study aimed to investigate the level of CD4^+^ T cells and forkhead box protein P3^+^ (FoxP3^+^) Treg cells, as well as T-cell-related-cytokines such as TNF-α, IFN-γ, IL-17A, and TGF-β1 in the peripheral blood of sarcoma patients. In addition, as there is a lack of studies concerning immune genes in the peripheral blood of sarcoma patients, we attempted to identify the genes related to Th differentiation in sarcoma patients, with a view of understanding the possible mechanisms that regulate Th and Treg cells in sarcomas.

## 2. Materials and Methods

### 2.1. Sarcoma Patients and Healthy Volunteers

**Figure 1:**
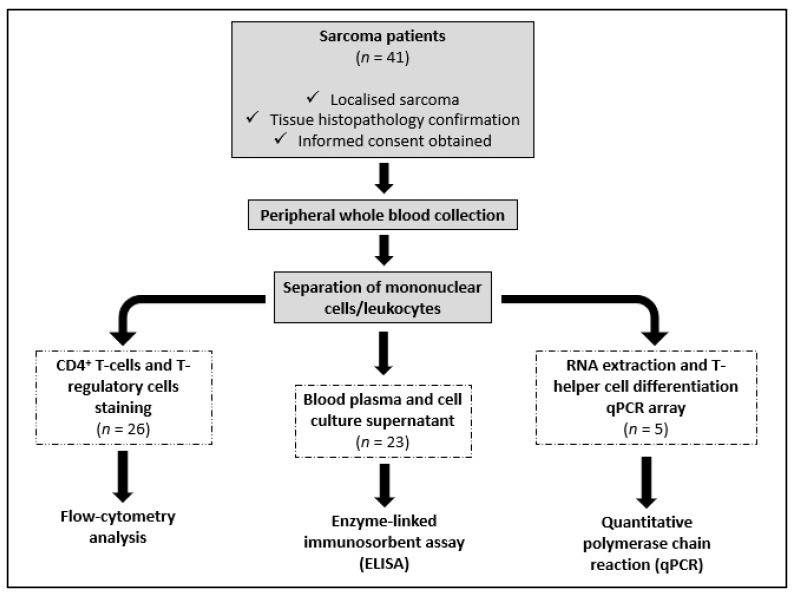
Flow chart of the research process.

This pilot study recruited 41 newly diagnosed sarcoma patients between 13 and 68 years old from the Orthopedic Oncology Unit, University of Malaya Medical Centre (UMMC), Kuala Lumpur, Malaysia. Following the inclusion criteria, only patients with localized disease were included in this study (Figure 1). This was determined at presentation via staging studies, which included a computer tomography of the chest, bone scan, and positron emission tomography (PET) scan. PET scans are a sensitive in detecting marrow involvement. Sarcoma patients with recurrent disease, carcinoma, and non-malignant tumors, and those with comorbidities such as diabetes, ischemic heart disease, and hypertension, which could have influenced the results, were excluded from this study. The diagnosis of sarcoma was confirmed via tissue histopathology. Blood samples were taken from each patient using a standard venipuncture technique before the commencement of treatment. Twelve healthy volunteers with no significant illness (diabetes, ischemic heart disease, and hypertension) and no effective medications (inflammatory and immunotherapy medications) served as normal controls. This study includes blood samples from 10 controls and 26 sarcoma patients to compare the percentage of CD4^+^ T cells and Treg cells in peripheral blood using flow cytometry analysis, and 9 controls and 23 sarcomas to measure the concentration of cytokines in the plasma and culture supernatant using enzyme-linked immunosorbent assay (ELISA) (Table 1). Blood samples from five soft-tissue sarcoma patients and five normal controls were included to identify any differentially expressed genes related to T-helper cell differentiation in peripheral blood using quantitative polymerase chain reaction (qPCR) (Table 1). For qPCR analysis, we selectively collected samples from the soft-tissue sarcoma subtype to minimize variability between the patients. This pilot study was conducted following the Declaration of Helsinki. Written informed consent was obtained from patients and volunteers following the UMMC Medical Ethics Committee’s (MEC) approval (MEC number: 848.16) (Figure 1). 

### 2.2. Analysis of CD4^+^ T Cells and T-Regulatory Cells in Peripheral Blood

Peripheral venous blood was collected in an 8 mL Beckton Dickson (BD) Vacutainer^TM^ mononuclear cell preparation tube (CPT^TM^) with sodium citrate (Beckton, Dickson & Company, Franklin Lakes, NJ, USA). This cell preparation tube contains a citrate anticoagulant with a Ficoll Hypaque density liquid and a polyester gel barrier that permits cell separation of mononuclear cells during centrifugation. Briefly, the tubes were centrifuged (1500× *g* for 20 min) to separate the peripheral blood mononuclear cells (PBMCs) from whole blood. The recovered PBMCs were washed twice with BD sheath fluid, and cells were recovered by centrifugation (250× *g* for 15 min). Then, the cells were resuspended in 1 mL of BD sheath fluid at a 1 × 10^7^ cells/mL cell density. A commercial FoxP3 staining kit (Beckton Dickson), which includes mouse anti-human CD25-APC, mouse anti-human CD4-FITC, and mouse anti-human FoxP3-PE, was used to stain the CD4^+^ T cells and Treg cells as recommended by the manufacturer (Beckton Dickson). Briefly, 100 µL of cells (1 × 10^7^ cells/mL) were aliquoted into 5 mL tubes and extracellularly stained with 20 µL of mouse anti-human CD4-fluorescein isothiocyanate (CD4-FITC) and mouse anti-human CD25-allophycocyanin (CD25-APC) antibodies, respectively. The cells were incubated in the dark at room temperature for 20 min and washed. Following this, the cells were permeabilized and intracellularly stained with 20 µL of mouse anti-human FoxP3 phycoerythrin (PE) for 20 min in the dark at room temperature and washed. Then, the cells were resuspended and fixed in 500 µL of BD CellFIX (Beckton Dickson). The cells were stored at 2 °C to 8 °C in the dark before cell acquisition within 24 h of staining. The corresponding controls were also prepared using MS IgG1 KPA ITCL APC MAB MOPC 21, FITC-labelled mouse IgG1, KPA ITCL, and PE-labelled mouse IgG1, k I/Ctrl (Beckton Dickson). The cells were quantitated using a FACS CANTO II flow cytometer (Beckton Dickson) and analyzed by BD FACSDiva software (version 6; Beckton, Dickson & Company, Franklin Lakes, NJ, USA). A minimum of 25,000 CD4^+^ T cells was acquired for data analysis for each sample.

### 2.3. Peripheral Blood Leukocyte Culture and Quantification of Cytokines

Peripheral blood (6 mL) was collected in BD Vacutainer^®^ Lithium Heparin tubes (Beckton Dickson). The blood was centrifuged (400× *g* for 10 min at 4 °C) to separate the plasma and the buffy coat. The plasma was aliquoted into sterile 0.5 mL microfuge tubes. The buffy coat was treated with red blood cell (RBC) lysis buffer (Intron Biotechnology Inc., Sagimakgol-ro, Joongwon-gu Seongnam-Si, South Korea) to lyse any residual red blood cells. Following this, the peripheral blood leukocyte cells (PBLs) were recovered by centrifugation (400× *g* for 10 min at 4 °C). The cells were resuspended in 2 mL of Roswell Park Memorial Institute (RPMI)-1640 media supplemented with glutamine and N-2-hydroxyethylpiperazine-N’-2-ethanesulfonic acid (HEPES) (Cellgro; Mediatech Inc, Manassas, VA, USA), 10% fetal bovine serum (FBS/GIBCO; Invitrogen, San Diego, CA, USA), penicillin, and streptomycin (GIBCO; Invitrogen, San Diego, CA, USA). Cell count was adjusted to 1.0 × 10^6^ cells/mL and 100 µL cells were added into wells of a 96-well flat-bottomed plate (Orange Scientific, Braine-l’Alleud, Belgium). Following this, the cells in each well were exposed to 10 µg/mL of Concanavalin- A (Con A), a mitogen, by adding 100 µL of the diluted Con A solution (20 mg/mL) (Sigma Aldrich, St. Louis, MO, USA). The plates were incubated at 37 °C in a humidified 5% CO_2_ incubator for 72 h. Following this, the cells from each well were harvested, and culture supernatant was collected by centrifugation (400× *g* for 10 min). The plasma and culture supernatant were stored at −80 °C until used for quantification of cytokines (IL-17A, IFN-γ, TNF-α and TGF-β1) that were produced using commercial ELISA kits as recommended by the manufacturer (ELISA Ready-set-Go!^®^ kit; eBiosciences, San Diego, CA, USA). 

### 2.4. RNA Isolation and Quantitative PCR Array 

Total RNA was extracted from PBLs using a commercial ribonucleic acid (RNA) extraction kit as recommended by the manufacturer (QIAGEN^®^ RNA Blood Mini kit, QIAGEN GmBH, Hilden, Germany). Firstly, the red cells were selectively lysed by adding 7.5 mL erythrocyte lysis (EL) buffer *(provided with the kit)* (QIAGEN, Germany) to 1.5 mL of freshly withdrawn whole blood. The mixture was incubated on ice for 15 min and cells were recovered by centrifugation (400× *g* for 10 min at 4 °C). The supernatant was discarded. The pellet, which contained the leukocytes, was washed twice with 3 mL of the EL buffer and recovered by centrifugation (400× *g* for 10 min at 4 °C), and the supernatant was discarded. Following this, the pellet that contained the leukocytes was subjected to RNA extraction as recommended by the manufacturer (QIAGEN, Germany). The purity, quality, and integrity of the extracted RNA was assessed using NanoDrop^®^ ND-1000 (NanoDrop Technologies Wilmington, DE, USA) and Agilent 2100 Bioanalyzer (Agilent Technologies Santa Clara, CA, USA). Following this, 0.5 µg of total RNA extracted was reverse transcribed to complementary DNA (cDNA). The first-strand cDNA synthesis was conducted using the RT^2^ First Strand Kit (QIAGEN, Germany). The cDNA was then mixed with RT^2^ SYBR^®^ Green Fluor qPCR Mastermix (QIAGEN, Germany). The resulting mixture was dispensed into the 96-well plate of a commercial human T-helper cell differentiation (RT^2^ Profiler qPCR array (PAHS-503)) (QIAGEN, Germany). This profiler qPCR array was annotated with 84 primers involved in human T-helper cell differentiation into specific effector cells. The qPCR array analysis was performed in an iQ5 Optical Module PCR Detection System (Bio-Rad, Hercules, CA, USA) as recommended by the manufacturer (QIAGEN), using the following cycling program: heating at 95 °C for 10 min, annealing of primers at 95 °C for 15 s followed by one minute at 60 °C, and a final extension at 55 °C for 60 s. This cycle was repeated 40 times. The array also employed various quality-control approaches, which included human genomic deoxyribonucleic acid (gDNA) contamination, reverse transcriptase control (RTC), and positive PCR control (PPC). The cycle threshold (C_T_) values of human gDNA for all samples tested were greater than 35, indicating no deoxyribonucleic acid (DNA) contamination in the RNA samples. The C_T_ values were analyzed using Web-based SABiosciences PCR Array Data Analysis software (version 3.5, QIAGEN GmBH, Hilden, Germany) (URL: www.SABiosceinces.com/pcrarraydataanalysis.php (accessed on 2 August 2017)). The C_T_ value of each gene was normalized to the geometrically averaged C_T_ value of two housekeeping genes automatically selected from the housekeeping panel in the PCR array: beta-2-microglobulin (*B2M*) and glyceraldehyde-3-phosphate dehydrogenase (*GAPDH*). The qPCR array analysis was repeated using five biological replicates from sarcoma patients and normal controls, respectively, as a minimum of at least two biological replicates was recommended from each group for comparison [24].

### 2.5. Statistical Analysis

The data obtained were analyzed using IBM SPSS Statistics 25.0 software (IBM Corp., Armonk, New York, NY, USA). All data obtained were tested for normality using the Shapiro–Wilk normality test. The data obtained for mean percentage of CD4^+^ T cells and Treg cells and for cytokine quantification were not normally distributed; therefore, the Mann–Whitney *U* test was used. Spearman’s correlation coefficient (*r*) was used to determine the correlation among cytokines and T cells in sarcoma patients. Data obtained from the T-helper cell differentiation array was performed using a web-based qPCR array data analysis (version 3.5, QIAGEN GmBH, Hilden, Germany) (URL: www.SABiosceinces.com/pcrarraydataanalysis.php (accessed on 2 August 2017)). The Kaplan–Meier method was used for survival analysis. A *p* value < 0.05 was considered statistically significant.

## 3. Results

### 3.1. Demographic Data 

Demographic and clinical characteristics of the sarcoma patients are summarized in Table 1 and Table 2, respectively. Based on sarcoma subtypes, 19 patients (46.34%) were diagnosed with bone sarcomas, and 22 patients (53.66%) were diagnosed with soft-tissue sarcomas. The majority of the sarcomas were located in the lower limb (33 patients (80.49%)), three patients had it located in the upper limb (7.32%), four patients in the chest (9.76%), and one patient in the back (2.44%). The sarcoma subtypes used for each analysis in this study are provided as (Supplementary Data Table S1). 

### 3.2. CD4^+^ T Cells and Treg Cells in Peripheral Blood

**Figure 2:**
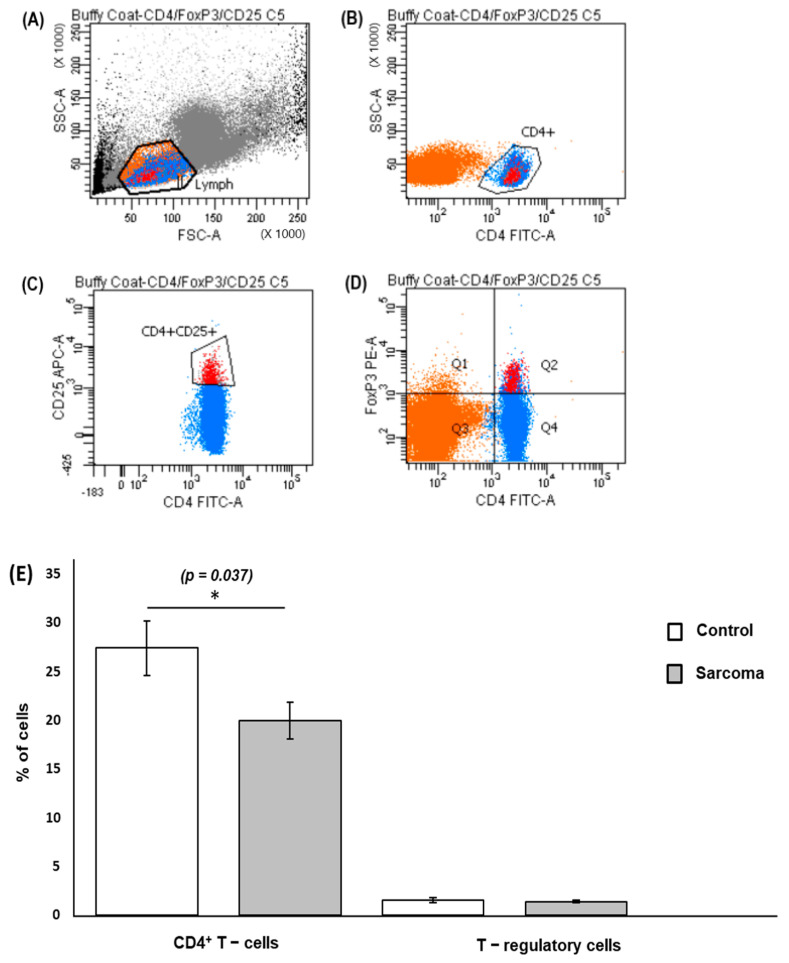
Gating strategy and flow cytometry quantification of CD4^+^ T cells and T-regulatory cells. PBMCs were extracellularly stained with FITC-conjugated anti-human CD4 antibody and APC-conjugated anti-human CD25 antibody and intracellularly stained with PE-conjugated anti-human FOXP3 antibody. (**A**) Lymphocyte population (labelled in orange) was gated from PBMCs following forward scatter (FSC) and side scatter (SSC) characteristics. (**B**) Gated lymphocytes were then separated into CD4^+^ T cells (labelled in blue). (**C**) CD4^+^CD25^+^ cells. (**D**) CD4^+^CD25^+^FoxP3^+^ cells (labelled in red, Q2). A minimum of 25,000 CD4^+^ T cells was gated for each sample during analysis. (**E**) Mean percentage of CD4^+^ T cells and Treg cells in sarcoma patients and normal controls. * Results show a significantly lower percentage (*p* = 0.037) of the CD4^+^ T cells in lymphocytes in sarcoma patients (20.18 ± 1.91%) compared to normal controls (27.76 ± 2.82%). The percentage of Treg cells in CD4^+^ T cells in sarcoma patients showed no significant difference (1.40 ± 0.12%) when compared to normal controls (1.53 ± 0.22%). For CD4^+^ T cells and Treg cells, data are represented as mean percentage of CD4^+^ T cells/T-lymphocytes ± standard error (SE) and Treg cells/CD4^+^ T cells ± standard error (SE), respectively.

The recovered PBMCs were stained with fluorochrome-conjugated antibodies to detect CD4^+^ T cells and Treg cells. Cells stained with FITC-conjugated anti-human CD4 were used to identify the CD4^+^ T cells and simultaneous staining with APC-conjugated anti human CD25 was used to identify the CD4^+^CD25^+^ cells. Staining with the Foxp3 transcription factor using an intracellular PE-conjugated anti-human Foxp3 antibody, which bound to the CD4^+^CD25^+^Foxp3^+^ T cells, was used to identify Treg cells. Doublets and singlets were excluded during the analysis (Supplementary Data; Figure S1). An example of the gating strategy used to isolate the CD4^+^ T cell and CD4^+^CD25^+^FoxP3^+^ T-cells is shown in Figure 2a–d. The CD4^+^ T cells were calculated as a percentage of the lymphocyte population and the Treg cells were calculated as a percentage of the CD4^+^ T-cell population in sarcoma patients or normal controls. The mean percentage of CD4^+^ T cells in the peripheral blood of sarcoma patients (20.18%) was lower (*p* = 0.037) than that of normal controls (27.76%) (Figure 2e). There was no difference (*p* = 0.633) observed between the mean percentage of Treg cells in sarcoma patients (1.40%) compared to normal controls (1.53%) (Figure 2e). The dot-point version of the bar chart is provided as Supplementary Data (Figure S2). No significant difference was observed (*p* = 0.080) for T-regulatory cells/T-lymphocyte population in sarcoma patients when compared to normal controls (Supplementary Data, Figure S3).

### 3.3. Cytokine Levels in Culture Supernatants and Plasma

**Figure 3:**
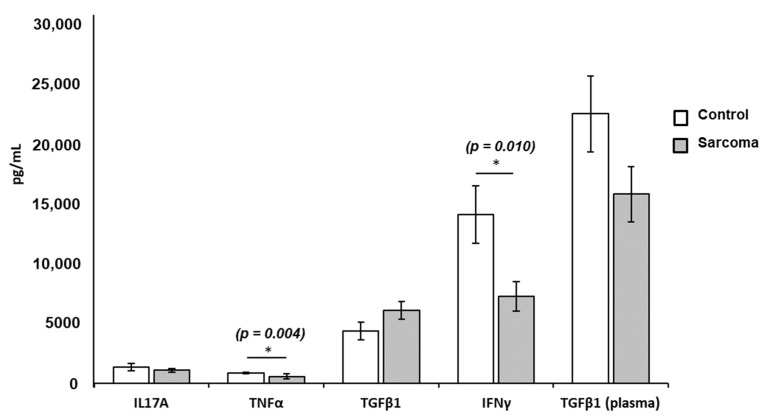
Mean concentrations of TNF-α, IFN-γ, IL-17A, and TGF-β1 in the PBL-stimulated culture supernatant and plasma levels of TGF-β1 from sarcoma patients and normal controls. Results show significantly lower levels of TNF-α (* *p* = 0.004; 562.87 ± 190.69 pg/mL) and IFN-γ (* *p* = 0.010; 7218.27 ± 1216.02 pg/mL) in sarcoma patients compared to normal controls (TNF-α = 843.74 ± 85.95 pg/mL; IFN-γ = 14,041.66 ± 2379.13 pg/mL). Each data point is represented as mean concentration of cytokines ± standard error (SE). The levels of IL-17A, TGF-β1, and plasma TGF-β1 did not show significance in sarcoma patients when compared to normal controls.

The level of TNF-α (*p* = 0.004) and IFN-γ (*p* = 0.010) in the Con-A-stimulated PBL culture supernatant from sarcoma patients was significantly lower than in normal controls (Table 3). There was no significant difference in the concentrations of IL-17A (*p* = 0.414) or TGF-β1 (*p* = 0.325) in the culture supernatants from the PBLs of sarcoma patients compared to normal controls (Figure 3). Plasma levels of TGF-β1 in sarcoma patients tended to be lower than the normal controls, but did not reach statistical significance (*p* = 0.098) (Figure 3). The concentration of other plasma cytokines, namely, TNF-α, IFN-γ, and IL-17A in sarcoma patients and normal controls, could not be determined as these cytokines were lower than the detection limit of the ELISA kits. The dot-point version of the bar chart is provided as Supplementary Data (Figure S4). 

### 3.4. Correlation among the Levels of Cytokines and T Cells 

The statistical correlations among the levels of cytokines and percentage of T cells (CD4^+^ T cells and Treg cells) in sarcoma patients are shown in Table 4. Two pairs showed significant positive correlation (IL-17A and TGF-β1, TNF-α and TGF-β1), for which the greatest correlation was observed between IL-17A and TGF-β1 (*r* = 0.580, *p* = 0.004). IFN-γ and CD4^+^ T-cell pairs showed significant negative correlation with *r* = −0.0486, *p* = 0.019. No correlations were observed among other pairs of cytokines or between cytokines and T cells.

### 3.5. Hematological Analysis

Of the 41 patients enrolled, we managed to obtain complete blood count results for only 34 patients. A total of 28 patients (82.35%) showed an alteration in their blood test results. Table 5 shows complete blood count results from patients with only an adult reference range (*n* = 33). Among the blood alterations observed in the patients were anemia with low hemoglobin (17 patients—51.51%), low red blood cells (11 patients—33.33%) and low hematocrit value (16 patients—48.48%), leukocytosis (15 patients—45.45%), iron deficiency anemia (10 patients—30.30%), and thrombocytosis (9 patients—27.27%). Only one female patient (aged 13) had a pediatric reference range, and all the full blood count parameters were within the reference range provided (not shown in the table). Of note, the differential counts of white blood cells were not available for all patients; therefore, it is not presented in this study.

### 3.6. Gene Expression Studies

**Figure 4:**
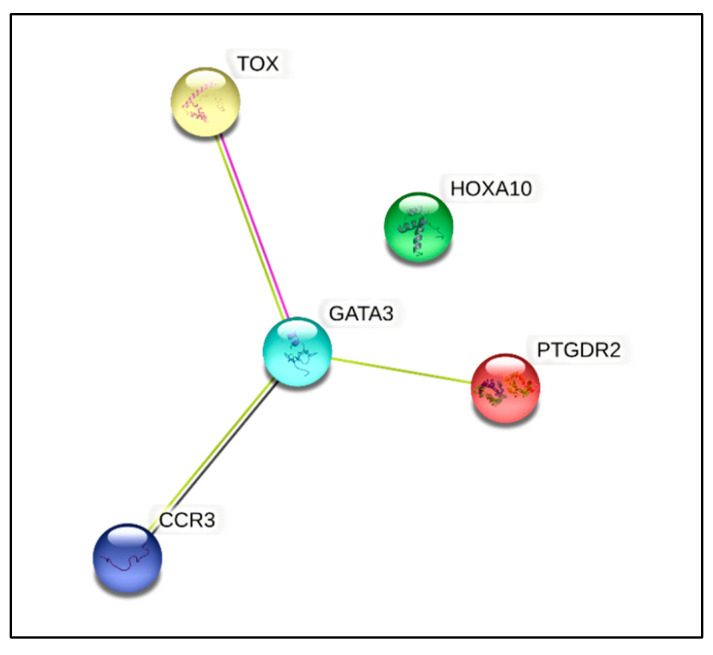
Protein interaction of significantly dysregulated genes. Protein–protein interaction and cluster analysis of the five differentially regulated genes from human T-helper cell differentiation array generated using the STRING Consortium 2022 software (version 11.5). There were five nodes and three edges. The PPI enrichment was found to be statistically significant (*p* < 0.000157).

The results from the qPCR analysis showed aberrant expression of 14 genes from 84 essential genes that were annotated in the commercial T-helper cell differentiation qPCR array used in this study (Supplementary Data; Figure S5). Of these 14 genes, the expression of five genes was shown to be significantly dysregulated (*p* < 0.05), where four genes (GATA binding protein 3 *(GATA3)* (NM_002051), thymocyte selection associated high mobility group box *(TOX)* (NM_014729), prostaglandin D2 receptor 2 *(PTGDR2)* (NM_004778), and C-C motif chemokine receptor 3 *(CCR3)* (NM_001837)) were found to be downregulated by two-fold (*p* < 0.05). These genes could be linked to various gene groups that included cytokine and receptors, Th2 subtype markers, transcriptional factors, and epigenetically regulated genes (Table 6). Only one gene (homeobox A10 *(HOXA10)* (NM_018951)), which was upregulated by two-fold (*p* < 0.05), belonged to two gene groups: the transcriptional factor and epigenetically regulated genes (Table 6). The Search Tool for the Retrieval of Interacting Genes/Proteins (STRING Consortium 2022) (Version 11.5) (https://string-db.org/ (accessed on 20 May 2022)) software was used to predict the protein–protein interaction (PIP) in a virtual situation to show the vital interactions as well as possible signaling pathways that these dysregulated genes affect [25] (Figure 4). 

### 3.7. Patient Prognosis

**Figure 5:**
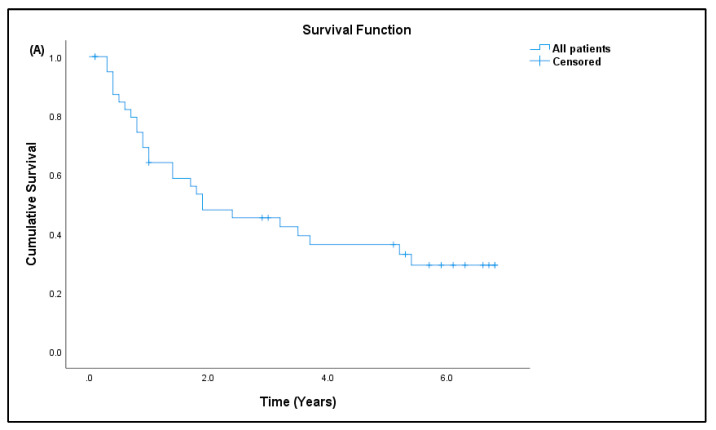

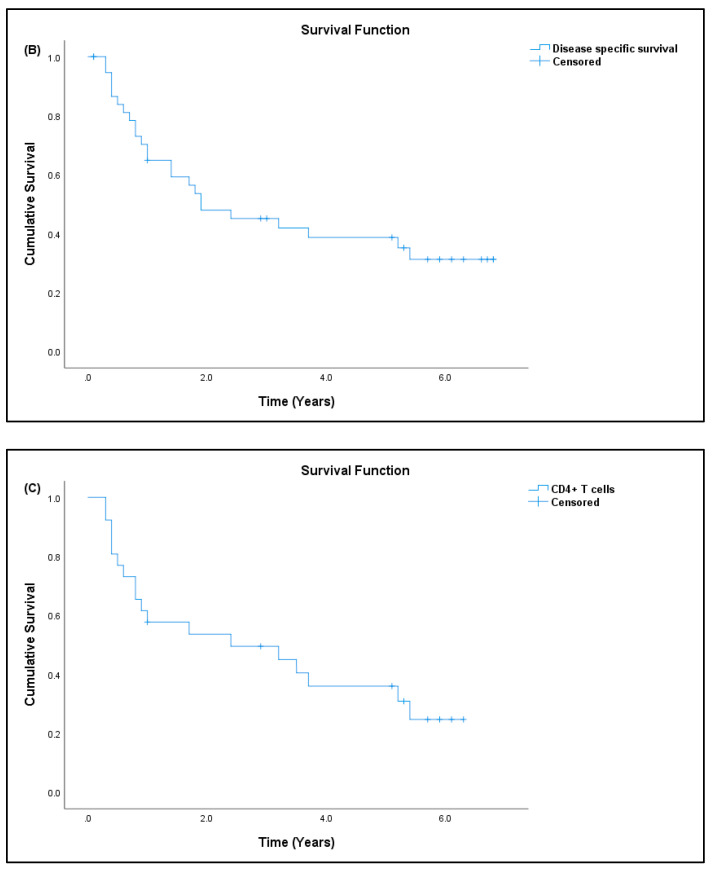
Kaplan–Meier curve for overall survival. (**A**) All sarcoma patients, (**B**) disease-specific sarcoma patients, and (**C**) patients tested for CD4^+^ T-cell/lymphocyte percentage.

**Figure 6:**
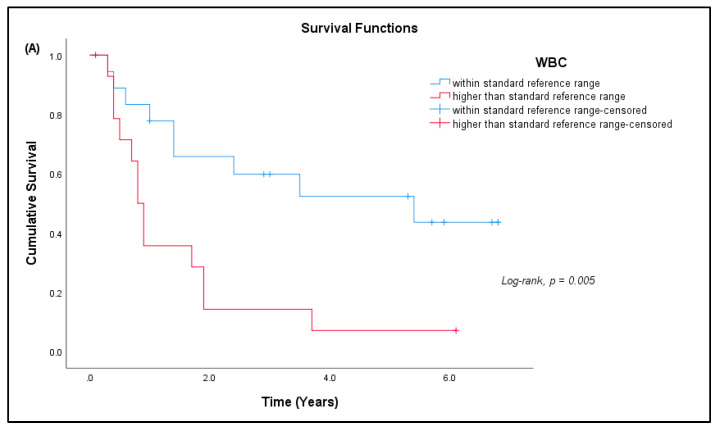

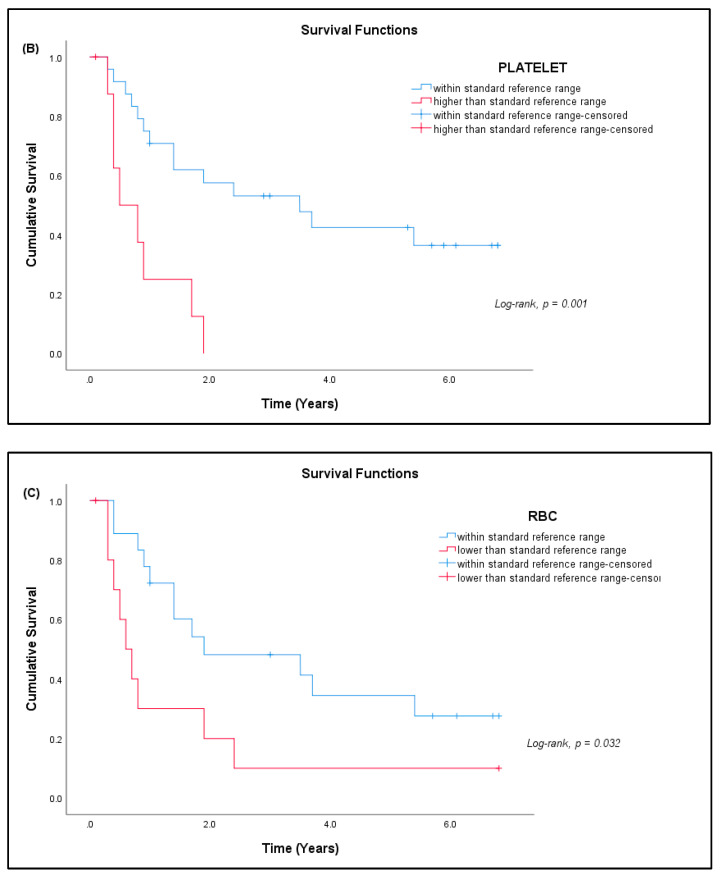
Kaplan–Meier curve for cumulative survival estimates among sarcoma patients by hematological abnormalities. (**A**) White blood cells (WBC), (**B**) platelets, and (**C**) red blood cells (RBC) when compared with the standard reference range (*n* = 33).

Out of 41 patients, 26 patients (63.41%) have passed away, out of which 24 (92.31%) died due to advanced disease, and two (7.69%) died due to other causes. Fifteen patients (36.59%) are still alive. Most patients survived for two years or less from the time of diagnosis (Table 7). The median survival rate of all sarcoma patients in this study was 1.9 years (95% CI: 0.17, 3.63), and the five-year survival rate was 36.6% (Figure 5a). The median for disease-specific survival for patients who died due to advanced disease was also 1.9 years (95% CI: 0.22, 3.58), with five-year survival at 38.5% (Figure 5b). The median survival for patients tested for CD4^+^ T cells was 2.4 years (95% CI: 0.00, 5.75), with five-year survival rate at 30.8% (Figure 5c). There was significant difference in the survival rate of sarcoma patients with hematological abnormalities of leukocytosis (*p* = 0.005), thrombocytosis (*p* = 0.001), and low red blood cells (*p* = 0.032) when compared to the standard reference range (Figure 6).

## 4. Discussion

In the recent years, there has been a growing body of evidence reported on significant roles of CD4^+^ T cells in tumor immunity. CD4^+^ T cells assist B cells, activate and expand CD8^+^ T cells, and generate and maintain memory CD8^+^ T cells [26,27]. Tumor-specific CD4^+^ T cells can reject tumor cells better than CD8^+^ T cells [28], as well as collaborate with NK cells to eradicate tumor [28]. There is also evidence to show that tumor-specific CD4^+^ T cells are directly involved in orchestrating tumor relapse and escape in-vivo [29]. On the contrary, increased numbers of another distinct subset of CD4^+^ T cells, Treg cells, exert suppressive effects in autoimmune diseases and allograft rejection, whereas a decreased number of these cells could enhance the immune response to cancers and chronic infections [30]. Previous studies have reported immune response in patients by evaluating T cells in peripheral blood leukocytes and cytokine measurement from mitogen-induced proliferative response of T cells [16,31]. Hence, this study was undertaken to compare peripheral CD4^+^ T cells and Treg cells and levels of a selection of T-cell-related cytokines in sarcoma patients with healthy controls.

CD4^+^ T cells are reported to play pivotal roles in fighting cancer progression via cytolytic activity or tumor microenvironment modulation [32]. In this study, the results show that the percentage of total CD4^+^ T cells was lower (*p* < 0.05) in sarcoma patients compared to healthy volunteers. This is worrying, as the main function of CD4^+^ T cells is to assist in the activation of antigen-specific CD8^+^ T cells [33]. In a cancer setting, CD4^+^ T cells orchestrate anti-tumor immune responses by activating CTLs in tumors via production of IL-2 and IFN-γ [26,34]. Moreover, CD4^+^ T cells also help to maintain CD8^+^ memory T cells in order to generate an effective secondary anti-tumor immune response [27,35]. Furthermore, reduced T-cell-mediated anti-tumor immune responses have been correlated with reduced circulating CD4^+^ T cells in nasopharyngeal carcinoma (NPC) patients [31]. In another study, a lower CD4^+^ T-cell count in venous blood was reported in patients with metastatic disease compared to those without metastasis [36]. Thus, a lower percentage of CD4^+^ T cells observed in sarcoma patients compared to normal controls also indicates reduced T-cell-mediated anticancer immune response in patients with sarcomas. Taken together, these findings are in line with the hypothesis that the reduction of CD4^+^ T cells can allow tumor advancement and reduce the survival of the host [26]. 

The regulation of immune response in sarcoma patients was also studied by measuring several cytokines (IFN-γ, TNF-α, IL-17A, and TGF-β1) produced by Con-A-stimulated T cells. There were reduced levels (*p* < 0.05) of IFN -γ and TNF-α from Con-A-stimulated peripheral blood lymphocytes from sarcoma patients compared to normal controls. The CD4^+^ T cells normally mediate anticancer responses by producing effector cytokines such as IFN-γ and TNF-α [33]. Furthermore, IFN-γ is the signature cytokine of Th1 immune responses, which plays a dominant role in cell-mediated immune responses needed to fight tumor progression [37]. IFN-γ also promotes the removal of tumor cells by impeding the activity of immune-suppressor cells, Treg cells, myeloid-derived suppressor cells, and tumor-associated macrophages [38,39]. Moreover, within the cytokine network of culture supernatant, TNF-α and IFN-γ can also be produced by CTLs [40], which can suppress cancer development [27]. CD8^+^ T cells are directly associated with tumor death and produce IFN-γ, which suppresses cancer development [19]. Reduced IFN-γ levels in sarcoma patients observed in this study may indicate reduced Th1 cells and CTL-mediated immune responses, which may allow tumor progression to occur. TNF-α, a pleiotropic cytokine [41] produced by activated macrophages and T-lymphocytes [20], induces production of Th1 cells [42]. Studies also show that TNF-α and IL-12 are co-stimulators for NK cells to produce IFN-γ [43], which is reported to work synergistically with TNF-α to generate reactive oxygen species and nitric oxide, which inhibit angiogenesis [33], activate macrophages and natural killer (NK) cells, and inhibit Treg cells [27,44], which suggests that these cytokines play substantial roles in cancer immunity. The reduced levels of TNF-α and IFN-γ in sarcoma patients observed in this study suggest that synergistic effects between these two cytokines are also reduced, which may support angiogenesis and tumor progression in these patients. 

Many studies have attempted to explore the immune-related genes in sarcomas at cellular levels [45,46,47]. In our study, we used a qPCR array annotated with primers related to T-helper-cell-differentiation genes to identify differentially regulated genes in leukocytes from peripheral blood that may be involved in the molecular mechanism and biological pathways that could modulate the peripheral immune response in sarcoma patients. *GATA3*, one of the differentially regulated genes, is a key transcription factor for Th2-cell differentiation [48], which inhibits Th1 differentiation via suppression of IL-2-STAT4 signaling pathway [49]. Abnormally low *GATA3* is also expressed in developing Th1 cells, which inhibits *IL-12Rβ2* expression following exposure to IL-12. Furthermore, ectopic *GATA3* expression also suppresses *STAT4* expression, thus failing to restore Th1 differentiation via *T-bet* upregulation [49]. The reduced *GATA3* expression in our study may explain the lower expression of *GATA3* on Th1 cells rather than on Th2 cells, which is evident with reduced IFN-γ production in sarcoma patients. Ectopic expression of *GATA3* in Th1 cells may also explain the failure to restore Th1 differentiation, thus reducing Th1 immune response in sarcomas. The *PTGDR2*, a chemoattractant receptor molecule *(CRTH2)*, and *CCR3*, are reported to be expressed on Th2 cells and eosinophils [50,51], and their co-expression was linked to Th2 response in sepsis inflammatory response [50]. The *HOXA10* gene, which is expressed on human progenitor CD34^+^ cells [52], is reported to be lower in mature PBLs [53]. Continuous over-expression of *HOXA10* in stem cells impedes development of common lymphoid precursor (CLP) cell differentiation into matured T, B, and NK cells, but supports myeloid differentiation [52]. Phosphorylation of tyrosine residues of *HOXA10* inhibits DNA binding upon interaction with IFN-γ signaling [54], and this may enable lymphoid differentiation [52]. In the present study, upregulation of the *HOXA10* gene in the PBLs may be due the expression of this gene in myeloid-derived cells. Moreover, the reduction in CD4^+^ T cells and IFN-γ production observed in this study could also indicate that phosphorylation of tyrosine residues did not occur in *HOXA10* to inhibit myeloid differentiation but impeded lymphoid differentiation in the sarcoma patients. Overall, sarcoma studies related to *PTGDR2*, *CCR3*, and *HOXA10* are still lacking. Therefore, further investigation of their potential role in the systemic immune response to sarcomas is warranted.

The *TOX* and its subfamily have been reported to be prognostic biomarkers in hematological malignancies and other cancers [55,56,57]. Increased *TOX* expression in PBLs from cancer patients was associated with reduced expression of anti-programmed cell death-1 (PD1) [58]. To the best of our knowledge, studies relating sarcoma and *TOX* are still underexplored. In an immune setting, the *TOX* gene is crucial to the development of T cells, NK cells, and lymphoid tissue inducers [59], as well as in the exhaustion of CD8^+^ T cells [58]. As such, the fundamental role of *TOX* in modulating the immune mechanism in sarcomas needs further validation.

The five-year overall survival rate for all patients, disease-specific survival, and patients tested for CD4^+^ T cells were 36.6%, 38.5%, and 30.8%, respectively. This finding is similar to a study that reported a five-year overall survival rate of 36% for retroperitoneal sarcoma patients [60]. The overall survival of patients with leukocytosis, thrombocytosis, and low red blood cells showed a significant difference when compared to patients within the standard reference range. This alteration in blood tests of sarcoma patients is similar to a study published by Rutkowski et al. [61], in which these common hematological abnormalities may correlate with prognosis [62,63] and metastasis [64] in cancer patients. Understanding immune cells’ overall outcome in sarcoma patients should be considered carefully. To our knowledge, there are many reports on extensive cell-based studies. Still, very few studies have focused on the immunological and molecular aspects of sarcomas based on peripheral immune cells. We recently came across a study investigating the peripheral immune status of sarcoma patients by evaluating 41 peripheral immune-cell subsets [65]. They observed improved disease outcomes in sarcoma patients and proposed that this may have been due to naturally developed antitumor immunity enhanced during disease progression. Yet, many sarcoma patients still succumb to recurrence and metastasis, which eventually reduces their overall survival. Our study showed reduced CD4^+^ T cells, IFN-γ, and TNF-α and identification of peripheral immune related genes in sarcoma patients compared to healthy controls, indicating reduced anti-cancer immunity in these patients. 

This study is a pilot project to investigate whether there are differences in the proportion of CD4^+^ T cells in sarcoma patients and healthy individuals using several research approaches. Although we were able to show that there is a reduction (*p* < 0.05) of CD4^+^ T cells in the peripheral blood of sarcoma patients compared to healthy volunteers, we acknowledge in retrospect that this study has several limitations. One of the major limitations is the small sample size, considering that sarcomas are heterogeneous tumors. A small sample size does not permit further evaluation of these findings with respect to histological subtype, age, and gender, which require a larger sample size and more effective clinical strategies. Furthermore, for the gene-expression studies, the buffy coat isolated from whole blood was used. The results may have been more meaningful if only CD4^+^ T cells isolated from the blood were used. In addition, the sample size used for the qPCR analysis was only a subset of the samples. Despite this, the expression of five genes was found to be significantly different between sarcoma patients and healthy volunteers. These five genes have been reported to have important role in various cancers, including sarcoma [48,58,66,67]. Further investigation on the expression of these genes may give clarity on these genes’ immune functions and mechanisms in sarcomas. This may add prognostic utility, leading to targeted immune therapies for sarcomas. 

## 5. Conclusions

In conclusion, the present study found that there was reduced levels of CD4^+^ T cells in peripheral blood, reduced levels of IFN-γ and TNF-α, and upregulation of the *HOXA10* gene in sarcoma patients compared to healthy volunteers, which suggests that sarcoma patients have reduced Th1 immune response, i.e., anticancer immunity. Sarcoma patients also appeared to have diminished Th2 immune response, as evidenced by the expression of Th2-related genes *GATA3*, *PTGDR2*, and *CCR3.* Moreover, downregulation of the *TOX* gene suggests a possible role in modulating anticancer immune responses in sarcomas. There is a need for more extensive studies on these genes, as the *PTGRD2*, *CCR3*, *TOX,* and *HOXA10* genes are still underexplored in sarcomas. Overall, there is a need to undertake further studies to evaluate potential genes and biomarkers that can lead to a better understanding of immune response on the prognosis of sarcoma patients in response to therapy.

## Figures and Tables

**Table 1:** Demographic profile of sarcoma patients and normal controls.

	Sarcoma	Normal Controls
**Flow cytometer analysis**	*(n = 26)*	*(n = 10)*
Age, median (IQR; range)	34.50 (32.75; 13–68)	33.00 (4.5; 23–40)
Female	13 (50%)	4 (40%)
Male	13 (50%)	6 (60%)
**Cytokine analysis**	*(n = 23)*	*(n = 9)*
Age, mean (SD; range)	35.74 (17.89; 15–68)	32.22 (4.84; 23–40)
Female	8 (34.78%)	3 (33.33%)
Male	15 (65.22%)	6 (66.67%)
**qPCR array**	*(n = 5)*	*(n = 5)*
Age, mean (SD; range)	38.80 (18.60; 16–62)	39.60 (12.30; 21–55)
Male	5 (100%)	5 (100%)
Female	-	-

IQR: inter quartile range; SD: standard deviation; qPCR: quantitative polymerase chain reaction.

**Table 2:** Clinical characteristic of sarcoma patients.

Clinical Features	Bone:	19 (100%)	Soft Tissues:	22 (100%)
**Type:**	Osteosarcoma	8 (42.11%)	Pleomorphic sarcoma	7 (31.82%)
	Chondrosarcoma	4 (21.05%)	Liposarcoma	5 (22.73%)
	Ewing’s sarcoma	3 (15.79%)	Synovial sarcoma	2 (9.09%)
	Pleomorphic sarcoma	2 (10.53%)	Leiomyosarcoma	2 (9.09%)
	Myofibroblastic sarcoma	1 (5.26%)	Rhabdomyosarcoma	2 (9.09%)
	Malignant GCT	1 (5.26%)	Desmoid tumor	1 (4.55%)
			Alveolar soft part sarcoma	1 (4.55%)
			Ewing’s sarcoma	1 (4.55%)
			Chondrosarcoma	1 (4.55%)
**Site:**	Femur	6 (31.58%)	Thigh	12 (54.55%)
	Pelvis	5 (26.32%)	Chest wall	4 (18.18%)
	Knee	3 (15.79%)	Pelvis	2 (9.09%)
	Fibula	2 (10.53%)	Leg	2 (9.09%)
	Scapular	1 (5.26%)	Back	1 (4.55%)
	Humerus	1 (5.26%)	Arm	1 (4.55%)
	Tibia	1 (5.26%)		

GCT: giant cell tumor.

**Table 3:** Summary of Con A stimulated cytokines in culture supernatant and plasma cytokine concentration in sarcoma patients and normal controls.

Cytokines	Normal Controls (*n* = 9) Mean ± SE (pg/mL)	Sarcoma Patients (*n* = 23) Mean ± SE (pg/mL)	*p*-Value
**IL-17A**	1320.87 ± 317.07	1066.58 ± 149.74	NS
**TNF-α**	843.74 ± 85.95	562.87 ± 190.69	0.004 *
**TGF-β1**	4338.48 ± 720.37	6042.73 ± 727.75	NS
**IFN-γ**	14,041.66 ± 2379.13	7218.27 ± 1216.02	0.010 *
**TGF-β1 (plasma)**	22,413.09 ± 3152.50	15,725.30 ± 2285.05	NS

*: *p* < 0.05; Con A: concanavalin A; IFN: interferon; IL: interleukin; NS: non-significant; TGF: transforming growth factor; TNF: tumor necrosis factor; SE: standard error.

**Table 4:** Correlation between cytokines from plasma, Con-A-stimulated peripheral blood leukocytes, and T cells.

Parameters		Cytokines (pg/mL) (*n* = 23)					T Cells (%) (*n* = 26)	
		IL-17A	TNF-α	IFN-γ	TGF-β1	TGF-β1 (Plasma)	CD4^+^ T-Cells	T-Reg Cells
**IL-17A**	*r*		0.039	0.264	0.580 **	0.39	0.055	0.209
	*P*		NS	NS	**0.004**	NS	NS	NS
**TNF-α**	*r*	0.039		−0.111	0.425 *	0.225	−0.272	0.207
	*P*	NS		NS	**0.043**	NS	NS	NS
**IFN-γ**	*r*	0.264	−0.111		−0.004	−0.127	−0.486 *	−0.079
	*P*	NS	NS		NS	NS	**0.019**	NS
**TGF-β1**	*r*	0.580 **	0.425 *	−0.004		−0.082	−0.014	0.407
	*P*	**0.004**	**0.043**	NS		NS	NS	NS
**TGF-β1 (plasma)**	*r*	0.039	0.225	−0.127	−0.082		−0.016	0.162
	*P*	NS	NS	NS	NS		NS	NS

*r*: Pearson’s correlation coefficient; *P*: *p*-value; NS: not significant; * *p* < 0.05; ** *p* < 0.01.

**Table 5:** Blood parameters of sarcoma patients.

Complete Blood Count (*N* = 33)				
Gender:		Male: 18	Female: 15	
Age: mean ± SD (range):		37.79 ± 18.09 (15–68 years)		
**Parameters**		**Reference range**	**Mean value ± SD**	**Status, *n* (%)**
WBC (× 10^9^/L)	**A**	4.0–10.0	9.82 ± 3.46	15 (45.45%) *
Platelets (× 10^9^/L)	**A**	150–400	332.64 ± 120.82	9 (27.27%) *
Hemoglobin (g/L)	**FA**	120.0–150.0	113.07 ± 18.84	1 (3.03%) *
	**MA**	130.0–170.0	135.33 ± 20.16	17 (51.51%) **
Hematocrit (L/L)	**FA**	0.36–0.46	0.34 ± 0.05	16 (48.48%) **
	**MA**	0.40–0.50	0.40 ± 0.06	
RBC (× 10^12^/L)	**FA**	3.80–4.80	4.19 ± 0.51	4 (12.12%) *
	**MA**	4.50–5.50	4.76 ± 0.63	11 (33.33%) **
MCV (fl)	**A**	77–97	83.00 ± 4.43	3 (9.09%) **
MCH (pg)	**A**	27.0–32.0	27.74 ± 2.29	10 (30.30%) **

A: adult; FA: female adult; MA: male adult; MCV: mean corpuscular volume; MCH: mean corpuscular hemoglobin; RBC: red blood cells; WBC: white blood cells. * Value higher than reference range (adult); ** value lower than reference range (adult).

**Table 6:** Dysregulated genes in the T-helper cell differentiation array compared with normal controls.

Genes	Descriptions	Fold Change	Fold Regulation	*p*-Value *
**HOXA10**	Homeobox A10	2.0905	Up	0.019
**CCR3**	C-C Chemokine receptor type 3	−3.1256	Down	0.045
**GATA3**	GATA binding protein 3	−3.4729	Down	0.021
**PTGDR2**	Prostaglandin D2 receptor 2	−4.7838	Down	0.015
**TOX**	Thymocyte selection-associated high-mobility-group box	−2.3072	Down	0.040

* *p*-value < 0.05.

**Table 7:** Survival outcome of sarcoma patients.

	Patients (N)	Percentage (%)
**# Survival outcome, *n* = 41 (100%)**		
Dead	26	63.41
Alive	15	36.59
**# Cause of death, *n* = 26 (100%)**		
Advanced disease	24	92.31
Others	2	7.69
**Survival duration, *n* = 24 (100%)**		
<6 months	6	25.00
≤1 year	7	29.17
<2 years	6	25.00
<5 years	3	12.50
>5 years	2	8.33

**#** Data-linkage service by the Biostatistics & Data Repository Sector, National Institute of Health, Setia Alam. **#** Mortality report from the National Registration Department (JPN).

## Data Availability

The data presented in this study are available on request from the corresponding author. The data are not publicly available due to ethical issue.

## Supplementary Materials

The following supporting information can be downloaded at: https://www.mdpi.com/article/10.3390/curroncol29080441/s1; Table S1: Sarcoma subtypes used in each analysis; Figure S1: Doublet and singlet exclusion in flow cytometry analysis; Figure S2: Dot plot for individual value of CD4^+^ T cells and Treg cells in sarcoma patients and normal controls; Figure S3: Graph of Treg cells/T-lymphocytes; Figure S4: Dot plot for individual values of IL-17A, TNF-alpha, TGF-beta1, IFN-gamma, and plasma TGF-beta1 cells in sarcoma patients and normal controls; Figure S5: Graph of log10 of normalized gene expressions between the standard control and sarcoma patients.
