# Supplementary material for: Immune Biomarkers in Blood from Sarcoma Patients: A Pilot Study
Source: Curr Oncol. 2022 Aug 5;29(8):5585–603. doi: 10.3390/curroncol29080441 (PMC9406743; doi:10.3390/curroncol29080441)
Supplement: Supplementary file 1 [file curroncol-29-00441-s001.zip › Figure S1 - proofread and revised.pdf]

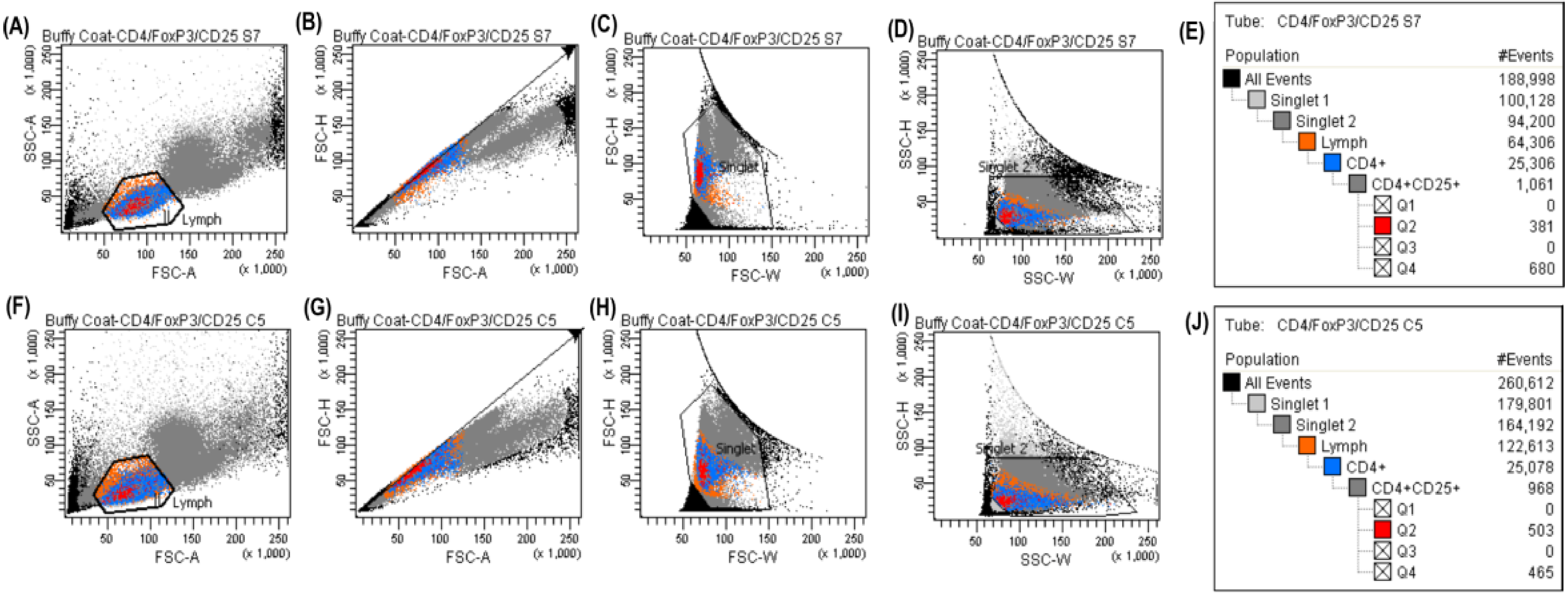

Figure S1: Doublets and singlets exclusion in flow cytometry analysis. Graphs above display events as a density plot (dot plots). Peripheral blood mononuclear cells (PBMCs) were sorted with forward scatter (FSC) and side scatter (SSC) to distinguish the lymphocytes population (labelled in orange) from monocytes and granulocytes (**A,F**). Cell debris and dead cells are at bottom left corner of the density plot. FSC-A vs. FSC-H were plotted for doublets exclusion (**B,G**). Singlets were distinguished from the PBMCs by FSC-W vs. FSC-H (**C,H**) and SSC-W vs. SSC-H (**D,I**). Figures (**A**), (**B**), (**C**) and (**D**) are representative density plot from a sarcoma sample and (**F**), (**G**), (**H**), and (**I**) are representative density plot from a normal control. Figures (**E**) and (**J**) are summary of number of events captured in the PBMCs.
