# Supplementary material for: Immune Biomarkers in Blood from Sarcoma Patients: A Pilot Study
Source: Curr Oncol. 2022 Aug 5;29(8):5585–603. doi: 10.3390/curroncol29080441 (PMC9406743; doi:10.3390/curroncol29080441)
Supplement: Supplementary file 1 [file curroncol-29-00441-s001.zip › Figure S3 - proofread and revised.pdf]

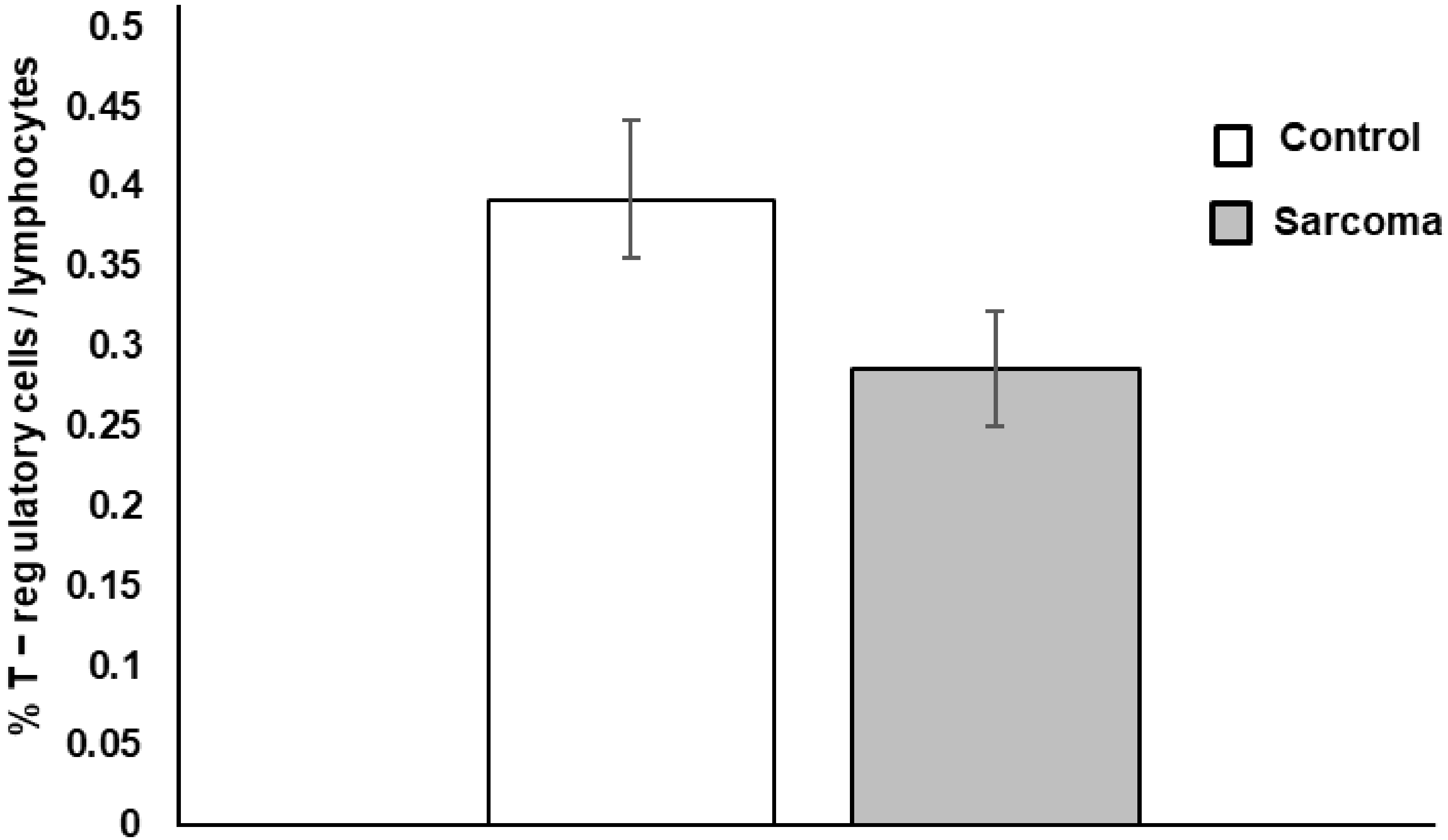

Figure S3: Graph of T-reg cells / T-lymphocytes. Treg cells were calculated as a percentage of the T-lymphocyte population in sarcoma patients or normal controls. There was no difference (p = 0.080) observed between the mean percentage of Treg cells / T-lymphocytes in sarcoma patients (0.29%) compared to normal controls (0.39%).
