# Supplementary material for: Immune Biomarkers in Blood from Sarcoma Patients: A Pilot Study
Source: Curr Oncol. 2022 Aug 5;29(8):5585–603. doi: 10.3390/curroncol29080441 (PMC9406743; doi:10.3390/curroncol29080441)
Supplement: Supplementary file 1 [file curroncol-29-00441-s001.zip › Figure S4 - proofread and revised.pdf]

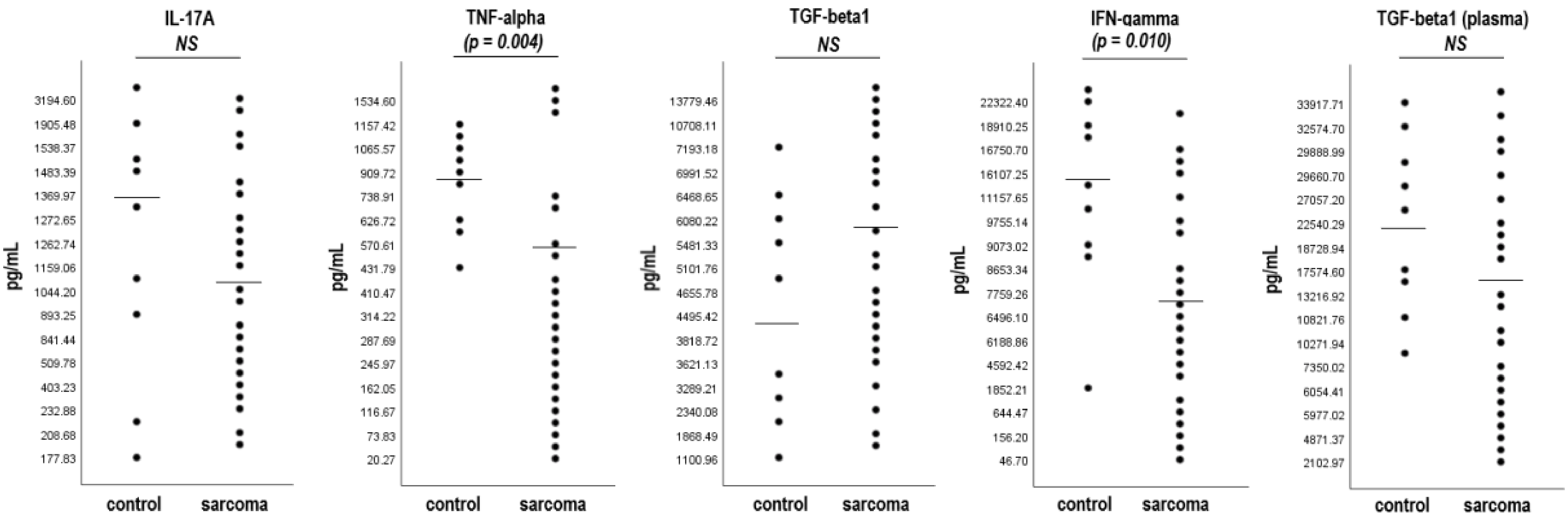

Figure S4: Dot plot for individual value of IL-17A, TNF-alpha, TGF-beta1, IFN-gamma and plasma TGF-beta1 cells in sarcoma patients and normal controls.
