# Supplementary material for: Immune Biomarkers in Blood from Sarcoma Patients: A Pilot Study
Source: Curr Oncol. 2022 Aug 5;29(8):5585–603. doi: 10.3390/curroncol29080441 (PMC9406743; doi:10.3390/curroncol29080441)
Supplement: Supplementary file 1 [file curroncol-29-00441-s001.zip › Figure S5 - revised.pdf]

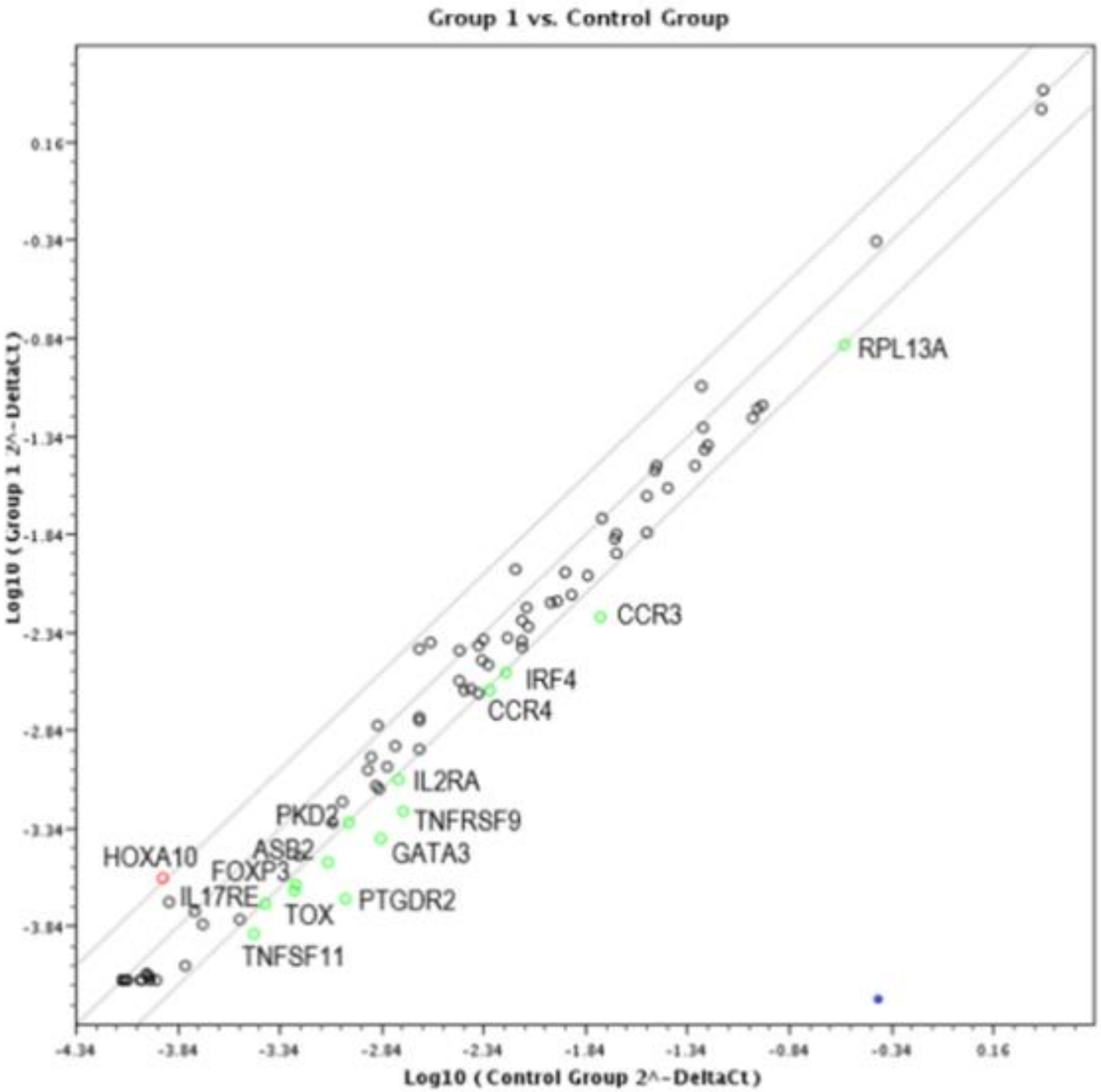

Figure S5: The graph of log10 of normalized gene expressions between the standard control and sarcoma patients. The graph show upregulated genes by two folds in red dots, and green dots represent downregulated genes by two-folds.
