# Supplementary material for: Immune Biomarkers in Blood from Sarcoma Patients: A Pilot Study
Source: Curr Oncol. 2022 Aug 5;29(8):5585–603. doi: 10.3390/curroncol29080441 (PMC9406743; doi:10.3390/curroncol29080441)
Supplement: Supplementary file 1 [file curroncol-29-00441-s001.zip › Table S1 - revised.pdf]

Table S1: Sarcoma subtypes used in each analysis .

**Analyses**	**Sarcoma subtypes (*n*)**	
**Flow cytometry analysis (*N* = 26)**	Osteosarcoma	*5*
	Chondrosarcoma	*5*
	Pleomorphic sarcoma	*3*
	Liposarcoma	*3*
	Synovial sarcoma	*2*
	Ewings sarcoma	*2*
	Rhabdomyosarcoma	*2*
	Desmoid tumor	*1*
	alveolar soft part sarcoma	*1*
	Myofibroblastic sarcoma	*1*
	Malignant GCT	*1*
**Cytokine quantification (*N* = 23)**	Pleomorphic sarcoma	*5*
	Osteosarcoma	*5*
	Chondrosarcoma	*3*
	Ewings sarcoma	*3*
	Liposarcoma	*1*
	desmoid tumor	*1*
	alveolar soft part sarcoma	*1*
	Rhabdomyosarcoma	*1*
	Leiomyosarcoma	*1*
	myofibroblastic sarcoma	*1*
	Malignant GCT	*1*
**Gene expression (*N* = 5)**	Pleomorphic sarcoma	*3*
	Leiomyosarcoma	*1*
	Liposarcoma	*1*

***[GCT;** giant cell tumour].*
